# Influence of Spatial Resolution on Space-Time Disease Cluster Detection

**DOI:** 10.1371/journal.pone.0048036

**Published:** 2012-10-24

**Authors:** Stephen G. Jones, Martin Kulldorff

**Affiliations:** 1 Department of Medical Informatics, BlueCross BlueShield of Tennessee, Chattanooga, Tennessee, United States of America; 2 Department of Population Medicine, Harvard Medical School and Harvard Pilgrim Health Care Institute, Boston, Massachusetts, United States of America; Kenya Medical Research Institute (KEMRI), Kenya

## Abstract

**Background:**

Utilizing highly precise spatial resolutions within disease outbreak detection, such as the patients’ address, is most desirable as this provides the actual residential location of the infected individual(s). However, this level of precision is not always readily available or only available for purchase, and when utilized, increases the risk of exposing protected health information. Aggregating data to less precise scales (e.g., ZIP code or county centroids) may mitigate this risk but at the expense of potentially masking smaller isolated high risk areas.

**Methods:**

To experimentally examine the effect of spatial data resolution on space-time cluster detection, we extracted administrative medical claims data for 122500 viral lung episodes occurring during 2007–2010 in Tennessee. We generated 10000 spatial datasets with varying cluster location, size and intensity at the address-level. To represent spatial data aggregation (i.e., reduced resolution), we then created 10000 corresponding datasets both at the ZIP code and county level for a total of 30000 datasets. Using the space-time permutation scan statistic and the SaTScan™ cluster software, we evaluated statistical power, sensitivity and positive predictive values of outbreak detection when using exact address locations compared to ZIP code and county level aggregations.

**Results:**

The power to detect disease outbreaks did not largely diminish when using spatially aggregated data compared to more precise address information. However, aggregations negatively impacted the ability to more accurately determine the exact spatial location of the outbreak, particularly in smaller clusters (<800 km^2^).

**Conclusions:**

Spatial aggregations do not necessitate a loss of power or sensitivity; rather, the relationship is more complex and involves simultaneously considering relative risk within the cluster and cluster size. The likelihood of spatially over-estimating outbreaks by including geographical areas outside the actual disease cluster increases with aggregated data.

## Introduction

Complete and timely reporting of infectious diseases is important for effective outbreak detection, which is in part based on statistically analyzing a study area for unexpected spatial and/or temporal clusters of cases. In the United States, case information used in surveillance efforts may come from various sources of administrative data, including health insurance plans, hospitals, emergency rooms, or physician offices, and can include identifiable patient information and clinical diagnostic detail. Studying disease clusters at the most precise level of spatial resolution, such as the patients’ address, is most desirable as this provides the actual residential location of the infected individual. However, this level of data precision is not always readily available or only available for purchase, and when utilized, it increases the risk of exposing protected health information (PHI) perhaps without the patient’s knowledge or consent. Also, added costs may be incurred when geocoding the relatively larger number of address-level cases, either monetary costs and/or resource costs due to extended processing time. Aggregating case data to less precise geographic scales may mitigate this risk and resource expenditure but at the expense of potentially masking smaller isolated high risk areas [1]–[6].

Underreporting of infectious diseases is a well-known issue that can also influence disease surveillance efforts. Health insurance plans could play a major role in the reporting of infectious diseases through submission of electronic administrative medical claims data to supplement passive physician reporting. These data are relatively easy and inexpensive to work with, and represent a volume rich source of persons diagnosed with infectious diseases [7]–[16]. Routinely collected electronic clinical data from the Harvard Pilgrim Health Care health maintenance organization (MCO) proved valuable in providing timely data for rapid disease surveillance, particularly for rare events and those without etiologic agent information [9], [11]. The Centers for Disease Control and Prevention (CDC) in collaboration with Harvard Medical School, Harvard Pilgrim Health Care and other nationwide health plans and their respective local health departments have incorporated electronic data from multiple administrative sources to detect localized outbreaks and facilitate rapid public health investigations [12]. When compared to state reported data, results suggest combining health plan data with the state could provide a more comprehensive view of certain infectious zoonotic disease clusters [13], [14]. Expanding data sharing efforts to include all communicable or infectious diseases for surveillance efforts would be ideal from a public health standpoint considering more than 250 million Americans have health insurance. Section 164.512(b) of the federal Health Information Portability Accountability Act (HIPAA) establishes safeguards allowing the disclosure of PHI without individual authorization for reporting of disease and vital events, and conducting public health surveillance, investigations and interventions. Depending on the exact intention of data sharing, other provisions could require signed agreements and legal review if being used for “health oversight” (section 164.512d), or independent review board approval may be required for research purposes (164.512i). While these provisions may ultimately permit the transfer of residential level case data, the inherent risks of such exchanges could be minimized if a less precise spatial resolution (*e.g.*, ZIP code, county) provided similar outbreak detection results.

Collecting case information at the residential street address level from administrative medical claims data is possible; however, if a less identifiable spatial resolution could successfully be used to estimate disease clusters, this would allow researchers to properly identify potential outbreaks while also masking the location identity of cases and thus protecting patient privacy. One approach to protecting patient privacy in spatial epidemiological research is geographical masking, which adds stochastic or deterministic noise to the original data matrix through modifying the geographic coordinates of the data points [1], [15]. However, a significant tradeoff may exist because increasing accuracy of results necessarily requires a mask with less introduced error, thereby directly increasing risk of patient exposure [16]. Another approach to protecting patient privacy is spatially aggregating data to a higher resolution (*e.g.*, from address to ZIP or county level). In general, the overwhelming majority of evidence purports the ability to detect outbreaks declines as spatial resolution declines, thereby providing researchers with objective evidence of weighing statistical precision against patient privacy concerns [4], [5], [6].

This study proposes to experimentally examine how spatio-temporal clusters of viral lung infections vary in statistical power and spatial precision as a function of spatial resolution. We focused on localized hot spot cluster detection via the Kulldorff scan statistic rather than global detection methods (*e.g.*, Tango's MEET). The scan statistic is commonly used in disease surveillance (*e.g.*, http://www.satscan.org/references.html) and has proven to have good statistical power for outbreak detection [17], [18]. Our works extends previous spatial-only efforts by utilizing the space-time scan statistic, simultaneously considering the variability in both cluster size and underlying relative risk using actual disease case information, and incorporates pragmatic spatial aggregations. Viral lung infections were chosen because a preliminary analysis indicated high case volume, a high degree of spatial and temporal variability across the study area and certain localized cluster spreading, thus making it a good candidate for experimental study. Additionally, respiratory infections are often utilized in syndromic surveillance research because they characterize many conditions of public health interest [4].

## Methods

### 2.1 Study Population

Administrative medical claims data were obtained retrospectively from commercial and government insured members of BlueCross BlueShield of Tennessee (BCBST), a large southeastern managed care organization. BCBST insures approximately 50% of the entire state’s 6Million+ population and adequately represents age, gender, income, and geographic distributions relative to the rest of the state. Ninety-three (93) percent of the state population lived in the same residence or same county as they did one year prior [19]. The study area consisted of the boundary of the state of Tennessee, USA and its surrounding counties.

### 2.2 Disease Episode Data

Within the health plan, all service claims (medical and pharmacy) are submitted to clinical grouper software which organizes the data into episode treatment groups (ETGs). An ETG is a basic illness classification methodology that provides a medically meaningful statistical unit representing a complete episode of care. Using the ETG methodology, baseline data consisted of viral lung infection episodes with and without comorbidities occurring from January 1, 2007–December 31, 2010 within the proposed study area collected from electronic administrative claims data. This produced 144042 unique viral lung infection episodes. International Classification of Diseases, Ninth Revision (ICD-9) diagnosis code *487.1* – *influenza with other respiratory manifestations* – occurred in an overwhelming majority (82.5%) of the viral lung ETGs. In addition to episode criteria, patient level information including a unique patient identifier code, residential street address, and episode start date (month/year) were also extracted.

Using a geographic information system (GIS) (Caliper Maptitude v5.0), all case records were geocoded to the street address level to obtain a geographical coordinate location (latitude, longitude). Approximately 85% (n = 122500) of the episodes had a valid geocoded location to their place of residence and were retained for further analysis. Of the 21542 non-geocoded cases, 39% were post-office box addresses, 5% had non-numerical street numbering, and the remaining 56% were unmappable due to other reasons (*e.g.*, street not found in GIS geocoder, symbolic characters in data).

We consulted with the BCBST internal IRB to determine if approval was needed for the use of the electronic data. This work did not disclose any PHI and had no human interaction; therefore, formal ethical approval from the IRB approval was not needed. Under HIPAA privacy section 164.512, no authorization from covered entities was required under the premise that this work is related to public health surveillance and thus obtaining consent was waived by the IRB as it was not applicable.

### 2.3 Space-time Permutation Scan Statistic

The space-time permutation scan statistic is described in detail elsewhere [17]. Briefly, a scan statistic is created by moving a cylindrical window over each county centroid, where the circular base represents a geographical area around a centroid and the cylinder height represents a time period. The cylinder is variable in both spatial size and temporal length. The method evaluates thousands of closely overlapping cylinders, each being a possible candidate for a disease cluster. Within each cylinder, the actual and expected number of disease cases, along with a Poisson generalized likelihood ratio (GLR) is calculated. Using Monte Carlo hypothesis testing [20], the maximum GLR from the actual data is compared to the maximum GLRs from each of 999 random simulated data sets generated under the null hypothesis. Relative risk (RR) for a significant cluster is calculated as the observed number of cases divided by the expected number of cases. For clusters where RR>1, this indicates the observed number of diseases cases is greater than expectation. Statistical significance is defined in terms of a p-value, and is computed as *p* = *R*/(*S*+1), where *R* is the rank of the GLR for the actual observation and *S* is the number of simulated data sets. Irrespective of the actual *P* value (*i.e.*, does not have to be below 0.05), the cluster with the highest *P* value is considered the primary cluster and all subsequent clusters in *P* value rank order are considered secondary. This analysis adjusts for any potential purely spatial and/or temporal variation, does not require a control comparison, and is most appropriate when interest is in space-time interaction, caused by for example a localized disease outbreak. It is not suitable for the detection of geographical clusters that are persistent over time, since those are adjusted away [17].

### 2.4 Simulated Data

We designed a simulation study to compare the ability to detect disease clusters at three levels of spatial resolution, the patient’s exact residential address location, versus the corresponding ZIP code and county centroids. Using address-level data, we created 10000 simulated datasets and inserted an artificial cluster into each one while varying cluster location, intensity (*i.e.*, relative risk) and cluster size across the datasets (Note: hereafter for clarity, any reference to a “simulated dataset” is to the creation of these artificially created cluster datasets, and does not pertain the 999 Monte Carlo simulations within the scan statistic methodology discussed below). A priori we determined each simulated dataset would contain a total of 1000 observations, which included both randomized observations (control cases) and the artificially created cluster of observations (treatment cases) discussed in detail below. The artificial cluster space was defined using a geometric square [5], [6] having an inscribed circle of radius *r*, therefore our term “radius” hereafter is for referential convenience. Simulation, rather than simply using the actual dataset one time, was necessary because multiple data runs are needed to generate a distribution of outcomes. Further, the simulation and insertion of artificial clusters provides a known outcome upon which detection rates are measured.

The following methods relate to creating SaTScan™ [21] case and geography files at the residential address level using a modified version of the macro accessory presented elsewhere [22]. We expand on previous work by determining the observation count within the artificial cluster as a function of cluster size and relative risk (RR) based on the underlying at-risk population, rather than using a fixed observation count and relative risk. To do so for each simulated dataset, we randomly selected the size of the cluster area by using a random number generator to select radius *r* between 1.6 and 48 km. The upper limit was chosen based on the size of the largest ZIP code and county within Tennessee, such that the largest possible artificial cluster could approximately overlap it completely in both latitudinal and longitudinal directions. We randomly placed the artificial cluster square within the study area and calculate *C*, the proportion of the at-risk population inside the area:
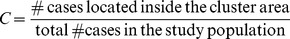



The value of *C* represents the baseline likelihood of a case being located inside the cluster area. A random relative risk (RR) value ranging from 1 to 10 was then derived and we simulated a space-time cluster by altering this likelihood based on increasing RR. We calculated *p*, the increase in probability of being inside the cluster given *C* and *RR* as:
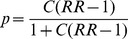
and *q*, the probability of being located outside the cluster given *p* as:



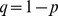
Thus, when RR = 1, *p* = 0 which intuitively indicates there is no increased risk of being located inside the cluster relative to outside the cluster. We derived *N_t_*, the number of treatment cases needed inside the artificial cluster given *C, RR,* and *p*, using the random variate value from a binomial distribution [Note: using SAS® v9.2, this was coded as *RANBIN*(0,1000, *p*)]. Last, we derived *N_c_*, the number of control cases needed outside of the cluster as:



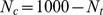



**Figure 1:**
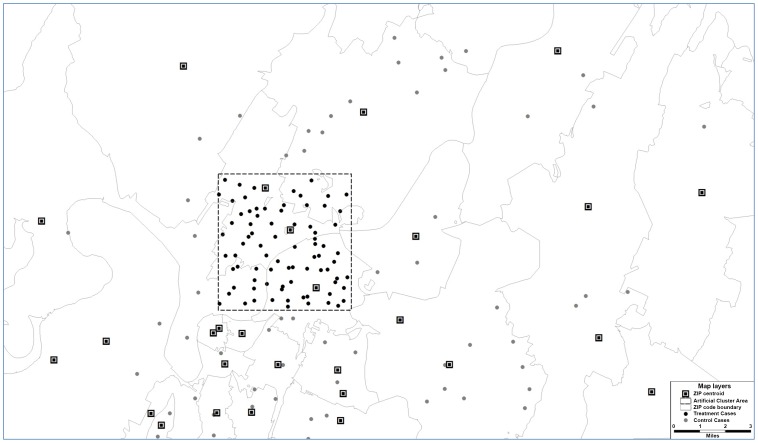
Example of a simulated dataset containing an artificially created infectious disease cluster. Example of a simulated dataset with an artificial cluster area (box) containing *N_t_* treatment cases (black dots) and surrounding *N_c_* control cases (grey dots) at the address level. Black squares with inscribed dots indicate ZIP code centroids to which address level cases would be spatially aggregated. Note: *N_c_* cases drawn from underlying population of cases to retain actual spatial location and event date is randomized. *N_t_* cases are randomly located within the cluster box area and event date is forced into a 3-month time period. Number of *N_t_* cases is calculated as a function of the underlying at-risk population within the cluster box area and randomly chosen relative risk value.

Note: We use the common “treatment-control” terminology here to represent typical experimental design comparisons. Simulations are created by applying a “treatment” to the cases by artificially placing them inside a block of space and time. This treatment effect is our “known” cases. The “control” cases are randomly dispersed and serve to fulfill the null hypothesis of the scan statistic, because it is known that no space-time interaction clusters will exist. In summary, “treatment” cases are inside the artificial cluster and “control” cases are outside the artificial cluster.

We randomly selected *N_c_* control cases from the underlying study population keeping their true geographical location, but for each one, we replaced the actual episode year-month date with a random year-month date within the 4-year study period drawing from the distribution of all cases. This data fulfills the null when the space-time permutation scan statistic is used, as it has no space-time interaction clusters. We randomly distributed *N_t_* treatment cases within the artificial cluster area using a random number generator applied to the bounding coordinates of the artificial cluster, replacing the actual date with a randomly assigned date within a 3-month period. This artificial cluster data fulfills the alternative when using the space-time permutation scan statistic (Figure 1).

### 2.5 Spatial Resolution

To simulate spatial aggregation, we replaced the specific geocoded address location with the latitude/longitude of the corresponding ZIP code and county centroid for each observation from the 10000 address level simulated datasets. This produced a total of 30000 simulated datasets: 10000 at the address level, 10000 at the ZIP code level and 10000 at the county level. We chose county and ZIP code level aggregations because these represent the commonly acquired and utilized areal units in disease surveillance activity.

### 2.6 Statistical Analysis

The free SaTScan™ software v9.1.1 [21] was used for all cluster detection analyses. Specific software settings included a retrospective space-time permutation probability model scanning for areas of high disease incidence, time aggregation of 1 month, a maximum spatial cluster size equal to 50% of the at-risk population, maximum temporal cluster size equal to 50% of the study period, a maximum of 999 Monte Carlo replications, and secondary clusters could not overlap other previously reported clusters. Statistical significance of spatial clusters is determined using α≤0.05.

For each spatial resolution level, we created 30 mutually exclusive groups based on each unique combination of 10 RR values (1–10) and 3 cluster radius size combinations (0–16 km, 17–32 km, 33–48 km). For interpretation ease, we hereafter refer to these geographical cluster sizes as small, intermediate, and large, respectively. We calculated four separate metrics to examine how spatial resolution of case information influences the ability to detect a disease outbreak across the gradient of RR values and cluster size. These four metrics included one measure of statistical power to detect a cluster irrespective of location, and three measures of spatial precision.

*Statistical Power* - The proportion of simulated datasets, under each RR||cluster size combination, for which a significant cluster was detected irrespective of it being the artificial cluster or not, represented as:
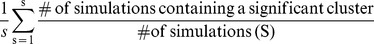



*Power with Spatial Precision (PSP)* – Similar to power, except the detected cluster must be sufficiently close in space to the artificial cluster. That is, the detected cluster was only recorded as successful if the distance between the detected cluster center was within one cluster radius of the true cluster center, represented as:
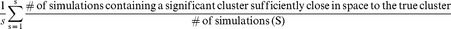
This means the detected cluster contains the center of the true cluster. Note that this metric is termed “power” by Ozonoff et al 2007 [5].

*Observation-level sensitivity (sensitivity)* – The proportion of the individual observations from the true cluster captured by the significant clusters, represented as:
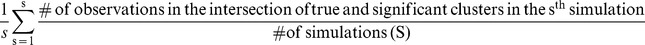



*Observation-level positive predictive (PPV) value* – The proportion of individual observations in the significant clusters belonging to the true clusters, represented as:
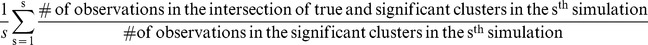
where *S* denotes the total number of simulated datasets (10000 for each spatial resolution in our case).

Sensitivity and PPV calculations follow that of others [23]. Note that under the null hypothesis when RR = 1, statistical power is the only metric that will have any measurable values because no spatial location requirements exist. A power equal to 5% is then expected.

## Results

**Figure 2:**
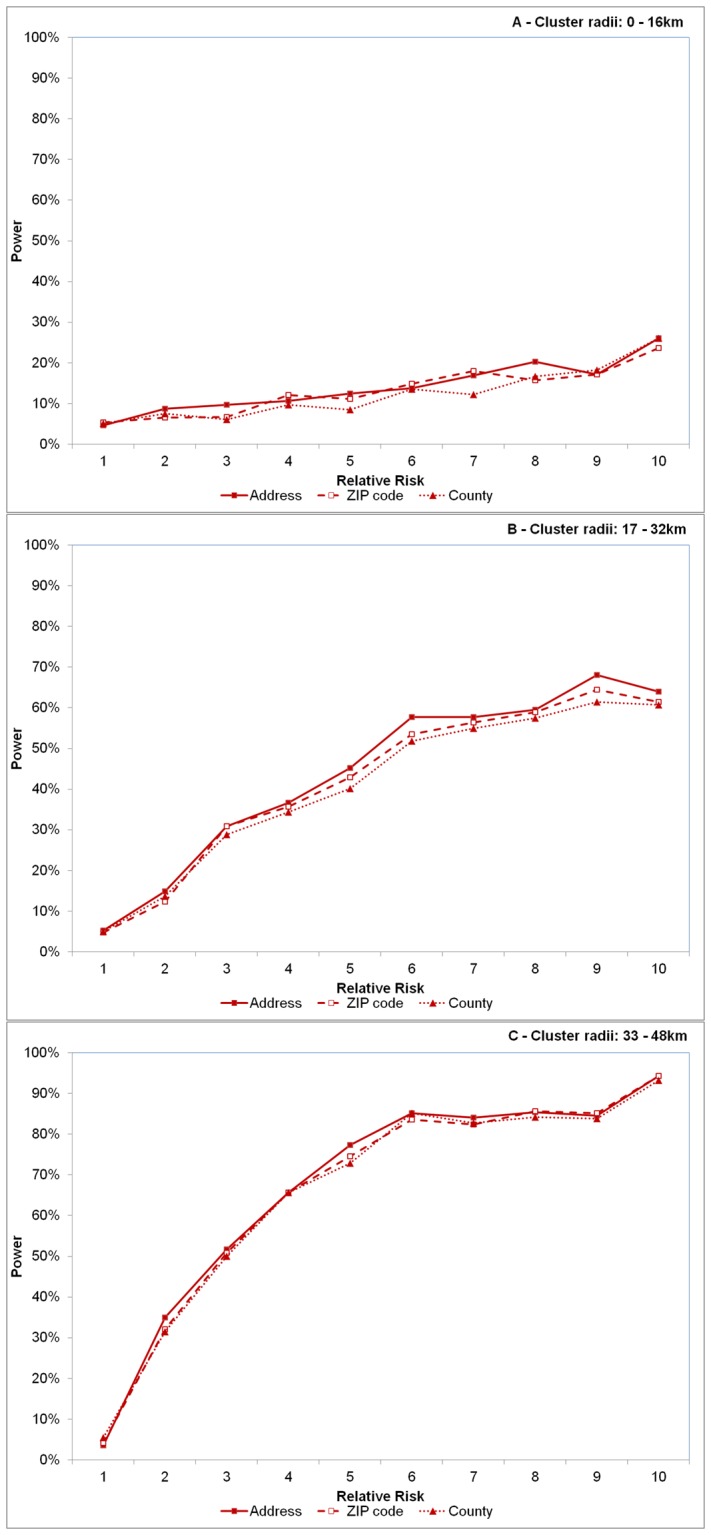
Effect of spatial resolution on power to detect significant space-time clusters. Effect of spatial resolution on power to detect significant space-time clusters at significance level α = 0.05 for varying sizes of cluster radii of 0–16 km (A), 17–32 km (B) and 33–48 km (C). Each line represents disease case data aggregated to different spatial resolutions – the address level (solid line with solid squares), the ZIP code level (dashed line with open squares) and county level (dotted line with triangles). Relative risk (abscissa x-axis) describes the intensity of the artificially created clusters, where RR = 1 indicates the risk of a disease case occurring inside the cluster area is equivalent to that of occurring outside the cluster area (see Fig. 1). RR = 10 indicates risk is 10 times higher inside the cluster area relative to outside the area.

The primary comparisons of interest were power to detect and spatial accuracy of the detected disease clusters measured as a function of spatial resolution, underlying relative risk and artificial cluster size. There were 617 different areal ZIP codes and 95 counties within Tennessee, with an overall average size of 107 km^2^ (sd = 98.2) and 709.8 km^2^ (sd = 239.7), respectively. A total of 10000 simulated runs were completed for each spatial resolution for a total of 30000 SaTScan™ runs (300000 case observations) generated from the 122500 actual viral lung infection cases containing valid geocoded locations. As relative risk increased, power to detect significant clusters also increased irrespective of artificial cluster size or spatial resolution (Figure 2). Power increased linearly as RR increased for small and intermediate sized clusters (Figure 2A, 2B), and increased rather exponentially for large clusters (Figure 2C). Power remained low for small clusters and never exceeded 26%. Overall, power declined approximately 1.4% and 2.0% on average when aggregating data to the ZIP code and county levels, respectively.

**Figure 3:**
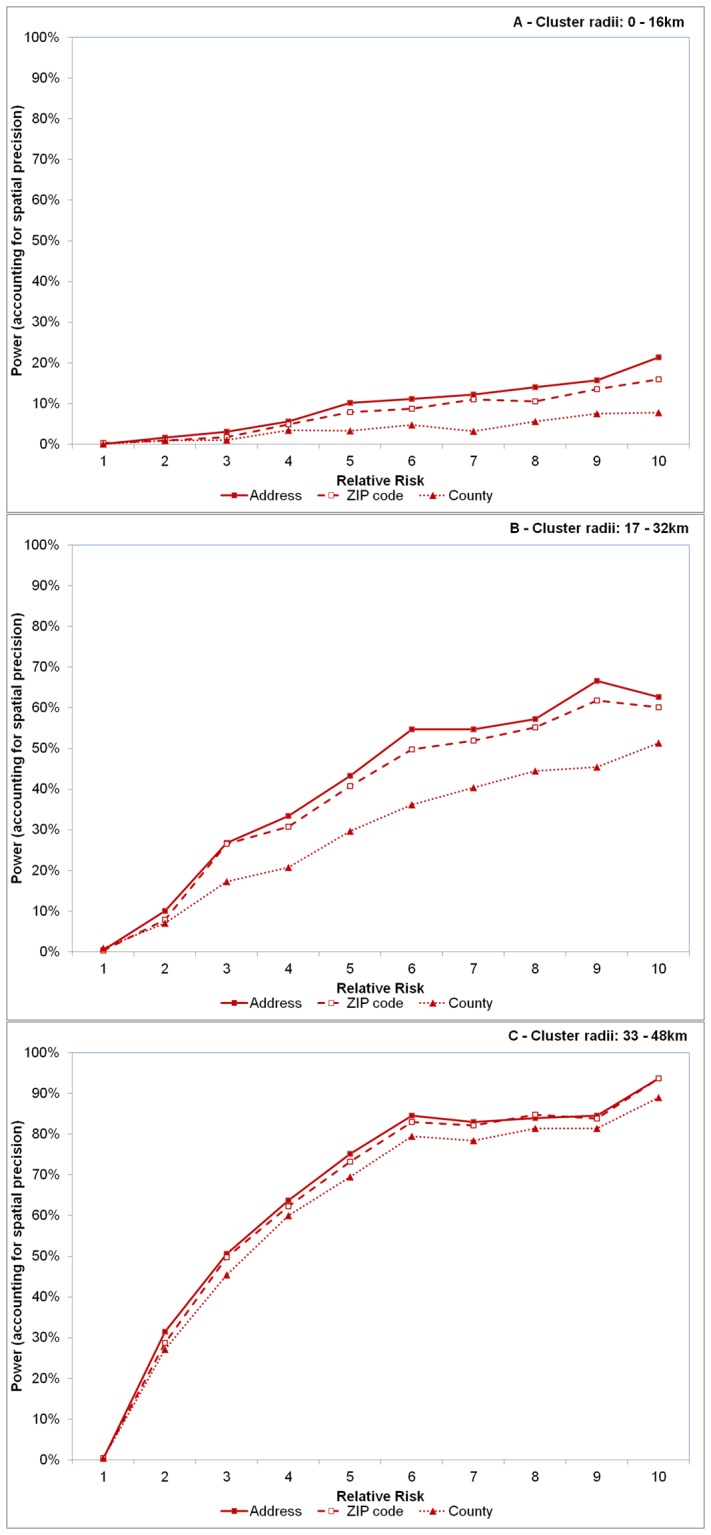
Effect of spatial resolution on power to detect significant space-time clusters accounting for spatial precision. Effect of spatial resolution on power to detect significant space-time clusters accounting for spatial precision for varying sizes of cluster radii of 0–16 km (A), 17–32 km (B) and 33–48 km (C). Each line represents disease case data aggregated to different spatial resolutions – the address level (solid line with solid squares), the ZIP code level (dashed line with open squares) and county level (dotted line with triangles). Relative risk (abscissa x-axis) describes the intensity of the artificially created clusters, where RR = 1 indicates the risk of a disease case occurring inside the cluster area is equivalent to that of occurring outside the cluster area (see Fig. 1). RR = 10 indicates risk is 10 times higher inside the cluster area relative to outside the area.

Power with spatial precision (PSP) followed a similar pattern as power. Here, ZIP code level values were only slightly lower than address level measures, though county level aggregations deviated comparatively more (Figure 3). Overall, PSP declined approximately 1.8% and 7.1%, on average, when aggregating data to the ZIP code and county level, respectively.

**Figure 4:**
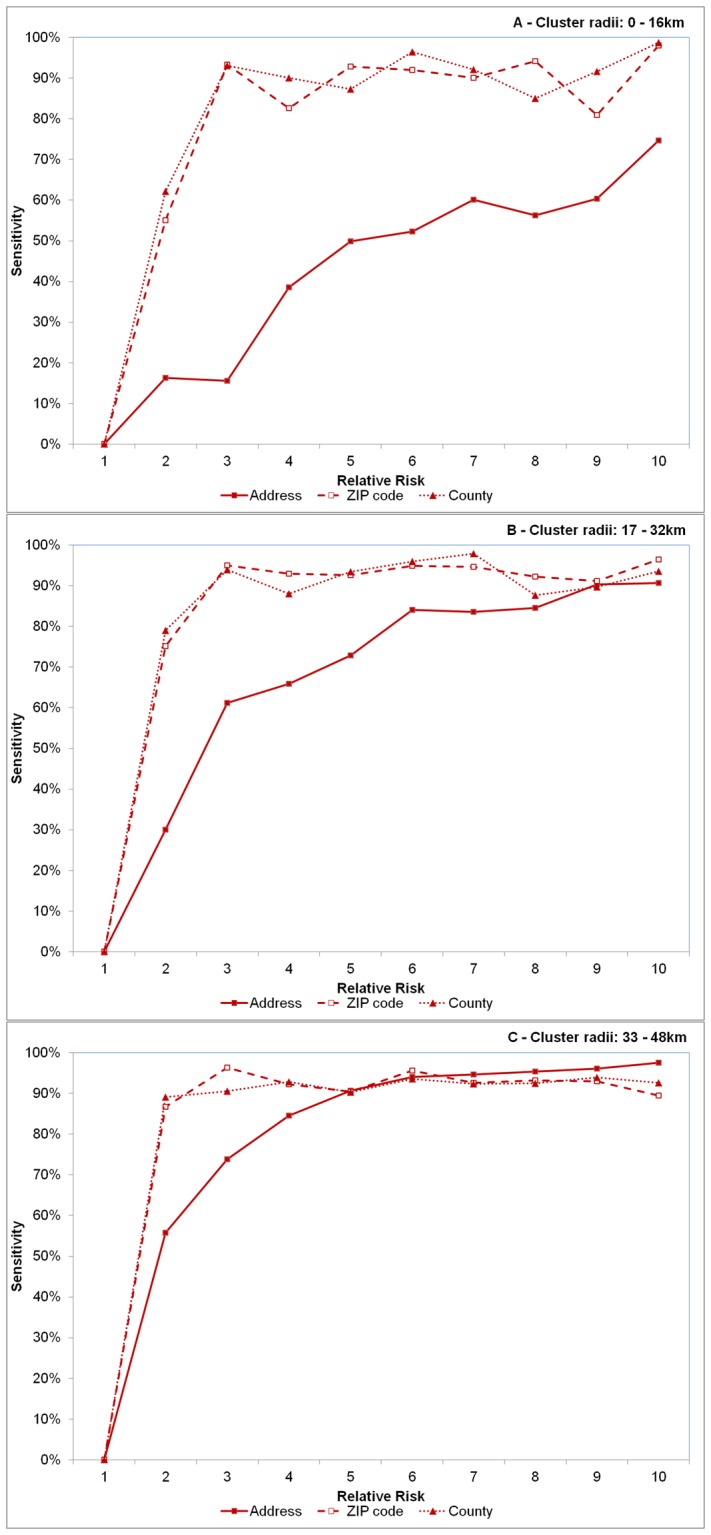
Effect of spatial resolution on sensitivity. Effect of spatial resolution on sensitivity, defined as the proportion of artificial observations included within the detected significant clusters for varying sizes of cluster radii of 0–16 km (A), 17–32 km (B) and 33–48 km (C). Each line represents disease case data aggregated to different spatial resolutions – the address level (solid line with solid squares), the ZIP code level (dashed line with open squares) and county level (dotted line with triangles). Relative risk (abscissa x-axis) describes the intensity of the artificially created clusters, where RR = 1 indicates the risk of a disease case occurring inside the cluster area is equivalent to that of occurring outside the cluster area (see Fig. 1). RR = 10 indicates risk is 10 times higher inside the cluster area relative to outside the area.

**Figure 5:**
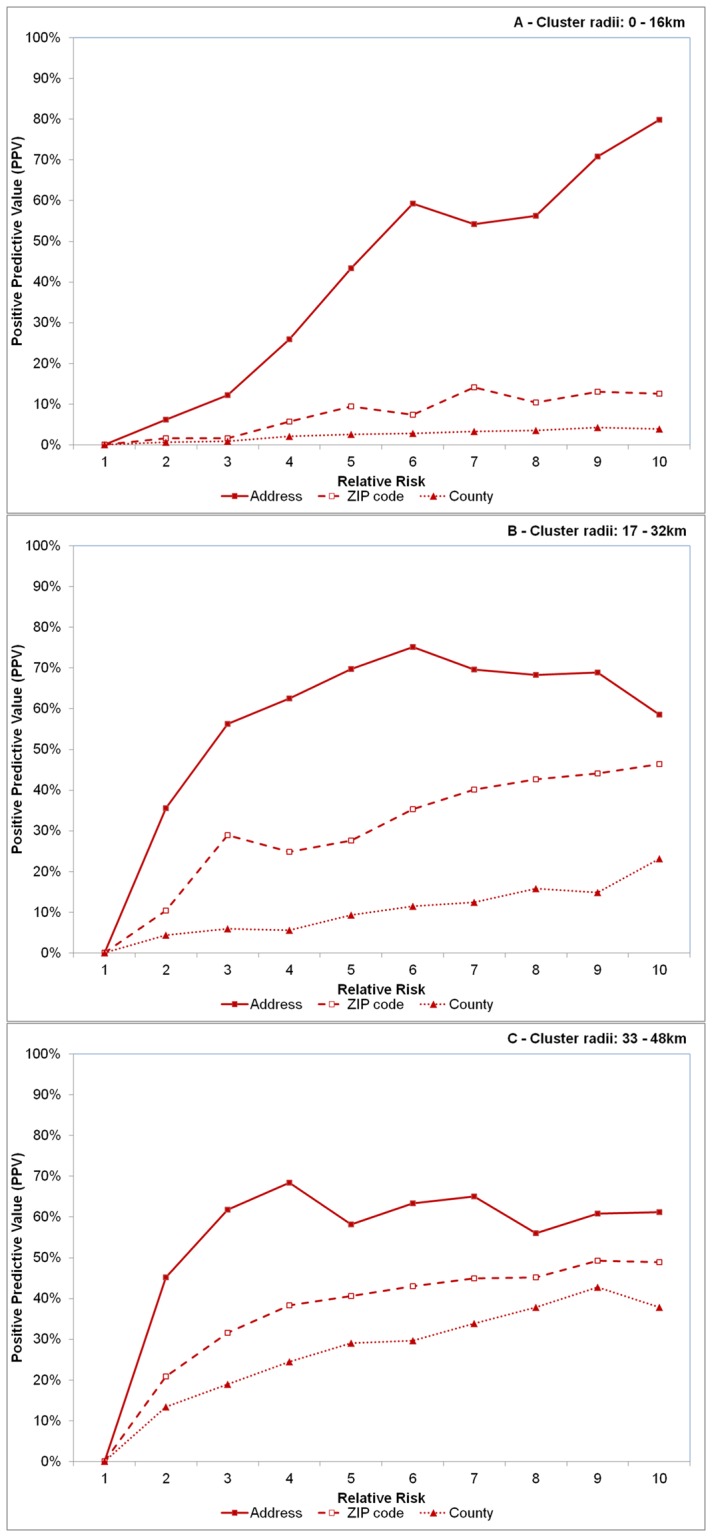
Effect of spatial resolution on positive predictive value (PPV). Effect of spatial resolution on positive predictive value (PPV), defined as the proportion of observations in the detected significant clusters and of the artificial cluster for varying sizes of cluster radii of 0–16 km (A), 17–32 km (B) and 33–48 km (C). Each line represents disease case data aggregated to different spatial resolutions – the address level (solid line with solid squares), the ZIP code level (dashed line with open squares) and county level (dotted line with triangles). Relative risk (abscissa x-axis) describes the intensity of the artificially created clusters, where RR = 1 indicates the risk of a disease case occurring inside the cluster area is equivalent to that of occurring outside the cluster area (see Fig. 1). RR = 10 indicates risk is 10 times higher inside the cluster area relative to outside the area.

In general, sensitivity was comparatively higher when case observations were recorded at the ZIP and county levels compared to the address across most RR and cluster size values. One obvious and notable deviation from this however was when clusters were large (32+ km radius) and RR>5 (Figure 4). Overall, sensitivity improved approximately 18.8% and 19.1% on average when aggregating data to the ZIP code and county levels, respectively. However, the opposite was observed for PPV; here, PPV was comparatively lower for spatially aggregated data and declined 25.8% and 37.3% on average for ZIP code and county level aggregations, respectively (Figure 5).

## Discussion

This study adds to the body of work examining the influence that spatial data aggregations have on detecting space-time clusters and accurately locating disease outbreaks. Our study is noteworthy because we derive observation counts within simulated clusters by incorporating relative risk calculations, versus a fixed observation count and risk level. We also vary artificial cluster sizes, use the actual underlying spatial distribution of cases within our simulation runs, and use pragmatic aggregation levels to further our effort of producing results that would more closely represent reality. This better allows for future research to benchmark against our findings, both in future simulation studies and real-world comparisons of actual observational findings. Finally, to the authors’ knowledge, all prior published studies in this area have involved spatial-only models, whereas we invoked a space-time permutation statistic to better simulate the true efforts of outbreak detection which involve both space and time determinations.

For the purposes of this paper, our discussion centers mostly on the comparisons of power and spatial accuracy as a function of spatial resolution versus the actual values independent of this scale comparison. The most noteworthy finding in our study is power to detect disease clusters does not diminish an appreciable amount when aggregating data to the less precise ZIP code level, though county level aggregations deviated most when clusters and RR were large. These relationships exist also when we require the detected cluster to be sufficiently close to the artificial cluster (Figure 3), though county level deviations were more evident here than in power calculations. More recent findings do however support our conclusion that spatial aggregations do not necessitate a loss of power [6], [24]; rather, the relationship is more complex as it involves simultaneously considering relative risk within the cluster and cluster size. This complexity is more prevalent in our sensitivity and PPV results discussed below.

Higher sensitivity values were recorded with aggregated data, irrespective of RR when cluster radius was less than 32 km, or when RR<5 for large clusters. Higher sensitivity was achieved, however at the expense of spatial precision, where PPV was lower for aggregated data irrespective of cluster size or relative risk. This trade-off between sensitivity and PPV commonly occurs in statistical comparisons because as the identifiable target area increases and captures more potential case observations (increased sensitivity), precision is inherently lost because more non-cases are also included (decreased PPV). For example, to identify all ZIP codes in the US with at least one case of Babesiosis (a very rare infectious tick-borne disease), simply include all ZIP codes in a list of potential sites to obtain 100% sensitivity. However, the level of precision would be much lower because many included ZIP codes do not contain the disease. The deviations in sensitivity and PPV were much more pronounced within small clusters and particularly more evident in county level aggregations. PPV remained very low (under 5%) for county level aggregations when cluster sizes were small and never exceeded 45% for the largest clusters.

Spatial aggregations appear to cause an over-estimation of the actual cluster area when relative risks are lower, particularly within smaller clusters, as noted by sensitivity and PPV outcomes. Intuitively, over-estimation can be expected when data are aggregated to coarser scales because there is a greater likelihood to encapsulate points that do not belong to the actual cluster. This happens because the growing scan statistic circle must travel a greater distance, relative to address data, to cover the respective ZIP/county centroids containing the cluster observations. Although there is a relatively short displacement distance incurred when “moving” a patient from their address to the ZIP code centroid in the study area [25], as the scan statistic circle grows, it will logically encapsulate more address observations not part of the original significant cluster area in order to reach the relatively more spatial disparate ZIP/county centroids. Thus, it could be expected that the actual radii of clusters from aggregated data will be larger compared to address level clusters. In fact, we observed this to be true where the average radii for ZIP and county level clusters were approximately 20% larger compared to address level. As the scan statistic grows to capture cluster observations, sensitivity increases but positive predictive values decreases. Thus, the ability to locate a larger number of observations contained in the outbreak improves using spatially aggregated data, but the locational certainty of the cluster diminishes. In practical terms, this means on-the-ground resources will be more likely to identify an outbreak if one occurs, though less likely to isolate it.

Our results are in direct contrast to others’ earlier findings [5], which report degradation in power and sensitivity when aggregating data to a more coarse scale. However, their study design was considerably different in that each simulated dataset contained 100 total observations with exactly 10 artificially embedded disease cluster points, having a rather fixed relative risk>10. Additionally, they utilized a spatial-only model with uniformed spatial distribution of points upon which to aggregate the data to simulate a reduction in spatial resolution. Therefore, it is expected that our findings which suggest a complex interaction between RR and cluster size would vary from the Ozonoff et al findings [5].

Our study is not without limitations. Artificial clusters arranged in squares do not necessarily represent the true spatial distribution of true outbreaks; however, this removes some of the potentially confounding interactions between cluster shape and outbreak detection methodologies. Further, the true disease cluster is not required to be circular to obtain good power [26]. Although we use a space-time permutation model, we did not vary the temporal length of the artificial cluster in this study due to the increasing level of permutations upon which to report on, and therefore this remains as an area needing further attention. We only report results for significant clusters defined using *p*≤0.05 and results could vary using other values; however, this is the most commonly used Type I error rate.

### Conclusions

When using the space-time permutation scan statistic, the ability to detect the presence of a significant disease outbreak does not largely diminish when using spatially aggregated data (i.e., ZIP or county level) compared to more precise address information. However, this data aggregation negatively impacts the ability to more accurately determine the exact spatial location of the outbreak. There is a greater likelihood of spatially over-estimating the outbreak and thereby including geographical areas that are not part of the actual disease cluster. The intent of disease surveillance and available/deployable resources for outbreak investigation will dictate whether this interchange between sensitivity and accuracy is appreciably large.
